# Aortopulmonary fistula following mitral valve replacement: a case report

**DOI:** 10.3389/fcvm.2025.1664133

**Published:** 2025-12-03

**Authors:** Nágila Simon Ziebell, Luiz Felipe Perin, Raul Rossi Filho, Estela Suzana Kleiman Horowitz, José Luiz da Costa Vieira, Fábio Vieira Caovilla, Rogerio Eduardo Gomes Sarmento Leite

**Affiliations:** Department of Cardiology, Instituto de Cardiologia (IC/FUC), Porto Alegre, Rio Grande do Sul, Brazil

**Keywords:** fistula, aortopulmonary, mitral valve, thrombosis, percutaneous treatment

## Abstract

Acquired aortopulmonary fistula is a rare condition that may cause heart failure symptoms secondary to high-output pulmonary overload. We report an unusual case of an aortopulmonary fistula secondary to an aortic injury sustained during mitral valve replacement, which was identified during follow-up because of recurrent heart failure symptoms and repeated hospital admissions. Multimodality imaging enabled precise characterization of the fistula. After a Heart Team discussion, percutaneous closure was selected as the preferred treatment strategy. Based on the assessment of the trajectory and morphology of the fistula, a patent ductus arteriosus (PDA) occluder was chosen. Although the ostium of the fistula was challenging to identify, successful closure was achieved using the PDA occluder.

## 1 Case report

The patient was a 58-year-old woman with a medical history of acute mitral valve insufficiency secondary to rheumatic fever, for which she underwent mechanical mitral valve replacement in 1989. Her cardiac history also included chronic atrial fibrillation. She had no other comorbidities and was under regular cardiology follow-up, receiving anticoagulation therapy with a coumarin derivative. Her long-term medications included a beta-blocker, a mineralocorticoid receptor antagonist, and a loop diuretic.

In January 2021, the patient was hospitalized for mechanical mitral valve thrombosis secondary to subtherapeutic anticoagulation, most likely due to poor treatment adherence. Given her young age and significant symptoms, the Heart Team decided to perform repeat surgery, replacing the mechanical valve with a bioprosthesis. The procedure was carried out under cardiopulmonary bypass; however, an accidental femoral artery decannulation occurred, requiring emergency ascending aortic cannulation. Despite this intraoperative complication, the surgery was successfully completed, and the patient experienced an excellent postoperative recovery, being discharged a few days later.

Over the following 24 months, the patient remained asymptomatic and continued to attend regular outpatient follow-up appointments. After this period, however, she sought emergency care at other hospitals due to episodes of decompensated heart failure, resulting in two hospitalizations. In 2024, upon evaluation at our reference center, she presented with congestive symptoms, and a continuous heart murmur was detected. Transthoracic echocardiography showed a left ventricular ejection fraction of 62%, a normally functioning mitral bioprosthesis, and tricuspid regurgitation with a pressure gradient of 29 mmHg from the right ventricle to the right atrium. Both ventricles appeared normal. However, in the parasternal short-axis view, a high-velocity regurgitant jet was identified between the ascending aorta and the right pulmonary artery, raising suspicion of an aortopulmonary fistula.

**Figure 1:**
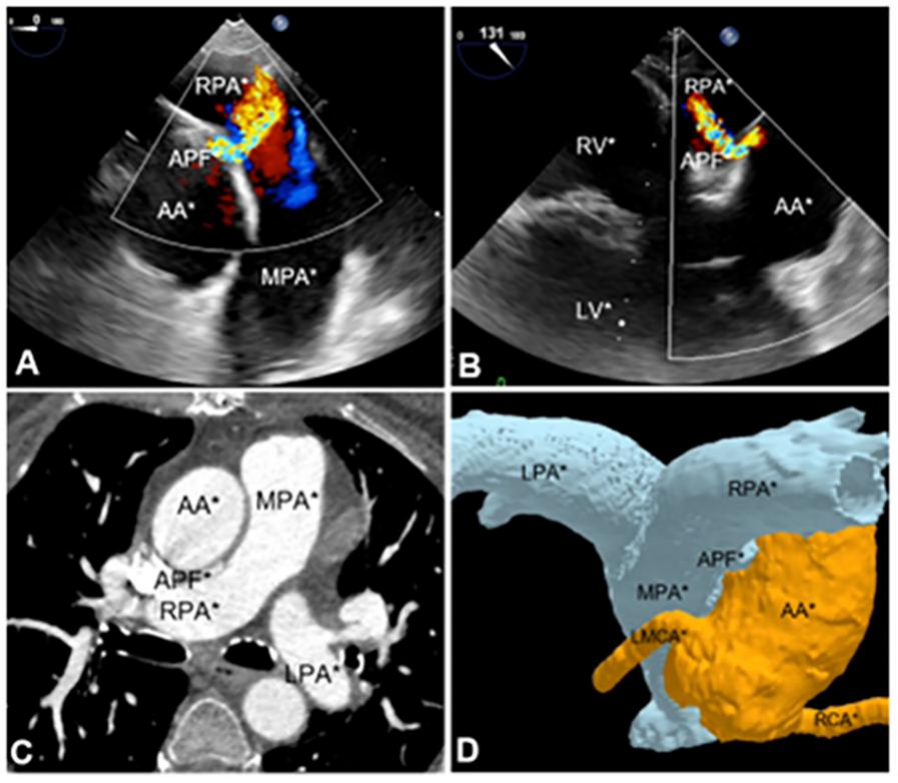
Imaging methods utilized for aortopulmonary fistula diagnosis. **(A)** TEE midesophagus ascending aorta short-axis section. **(B)** Midesophageal long-axis section. **(C)** Angiotomography of aortic and pulmonary vessels. **(D)** 3D angiotomography reconstruction. APF, aortopulmonary fistula; AA, ascending aorta; MPA, main pulmonary artery; LPA, left pulmonary artery; RPA, right pulmonary artery; LMCA, left main coronary artery; RCA, right coronary artery; RV, right ventricle; LV, left ventricle.

Further investigation with transesophageal echocardiography (TEE) confirmed the presence of the fistula, which measured 2 mm at the aortic orifice (Figures 1A,B and Supplementary Videos S1, S2), located approximately 30 mm above the aortic valve plane. In the midesophageal long-axis view of the ascending aorta, the maximum pressure gradient across the fistula was 78 mmHg. This resulted in high pulmonary output, with an estimated pulmonary artery systolic pressure of 44 mmHg.

Subsequently, computed tomography angiography (CTA) with 3D reconstruction of the aortic and pulmonary vessels was performed to determine the optimal correction strategy and assess the distance between the fistula and the coronary arteries (Figures 1C,D and Supplementary Videos S3). CTA revealed an aortopulmonary fistula located 26 mm above the aortic root and 38 mm below the brachiocephalic artery. The fistulous tract measured 10 mm in length, with an aortic orifice measuring 3 × 2 mm and a pulmonary orifice measuring 6 × 2 mm.

Based on these findings, the Heart Team decided to proceed with percutaneous closure of the fistula using an occlusion device. The choice of the device was guided by the tunnel-like path of the fistulous tract, the size of the orifice, and the length of the tract. Therefore, a PDA occluder was elected as the most suitable option to ensure optimal sealing and minimize the risk of peridevice leak.

**Figure 2:**
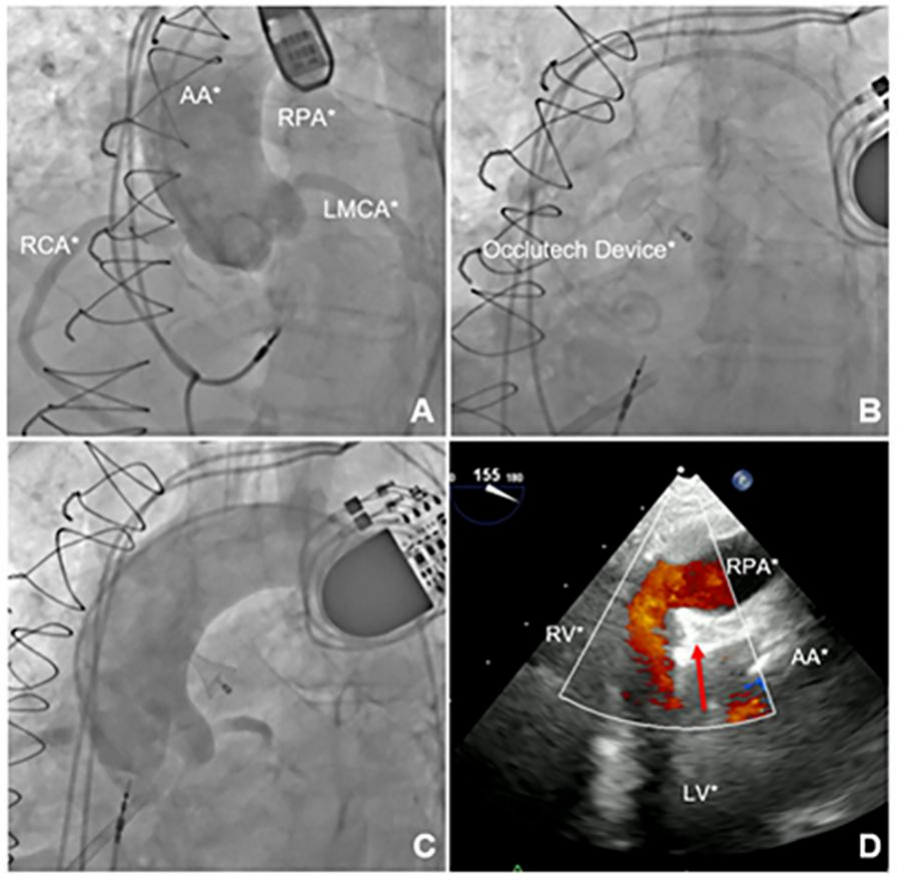
Imaging of the aortopulmonary fistula correction procedure. **(A)** Aortography demonstrating contrast flow to the right pulmonary artery. **(B)** PDA occluder deployed. **(C)** Control aortography demonstrating absence of flow to the right pulmonary artery. **(D)** TEE confirming absence of flow between the aorta and right pulmonary artery with the device successfully deployed (red arrow). AA, ascending aorta; MPA, main pulmonary artery; LPA, left pulmonary artery; RPA, right pulmonary artery; LMCA, left main coronary artery; RCA, right coronary artery; RV, right ventricle; LV, left ventricle.

The procedure was performed through a right common femoral artery puncture. The arterial approach was preferred because accessing the fistula from the higher-pressure aortic side toward the lower-pressure pulmonary artery was technically more feasible. A 6-Fr pigtail catheter was used for aortography to identify the fistula and determine its location, although its exact location could not be precisely identified (Figure 2A and Supplementary Video S4).

Attempts at selective catheterization under TEE guidance were made using 6-Fr Judkins Right (JR) 4.0, 6-Fr Multipurpose 2.0, and 6-Fr Amplatz Left 1.0 catheters. However, these attempts were unsuccessful, likely due to the high and anterior position of the fistula, which made it difficult to align the catheters with the fistulous orifice. Thus, puncture of the right radial artery provided a more favorable catheter angle within the ascending aorta, allowing the fistula to be successfully crossed using a 6-Fr JR 4.0 catheter and a 0.014-inch guidewire. Nevertheless, selective catheterization remained challenging due to the difficulty in precisely identifying the fistulous opening.

After crossing the fistula with the JR catheter, a long 0.035-inch hydrophilic guidewire was advanced into the right pulmonary artery and further into the inferior vena cava, where it was snared using an endovascular Snare system through the right femoral venous access. This approach enabled the introduction of a 7-Fr delivery system via the venous access, through which the Occlutech PDA Occluder 8/10 was successfully deployed from the pulmonary artery toward the aorta. This strategy allowed for optimal sealing of the aortic–pulmonary communication while maintaining delivery through the venous access.

The device was successfully deployed in an optimal position, achieving complete closure of the fistula (Figures 2B,C and Supplementary Video S5). Postprocedural TEE confirmed the absence of peridevice leaks (Figure 2D and Supplementary Video S6). The patient was discharged with significant improvement in symptoms, no longer requiring diuretic therapy, and was transitioned to a direct oral anticoagulant due to poor adhesion to coumarin.

**Figure 3:**
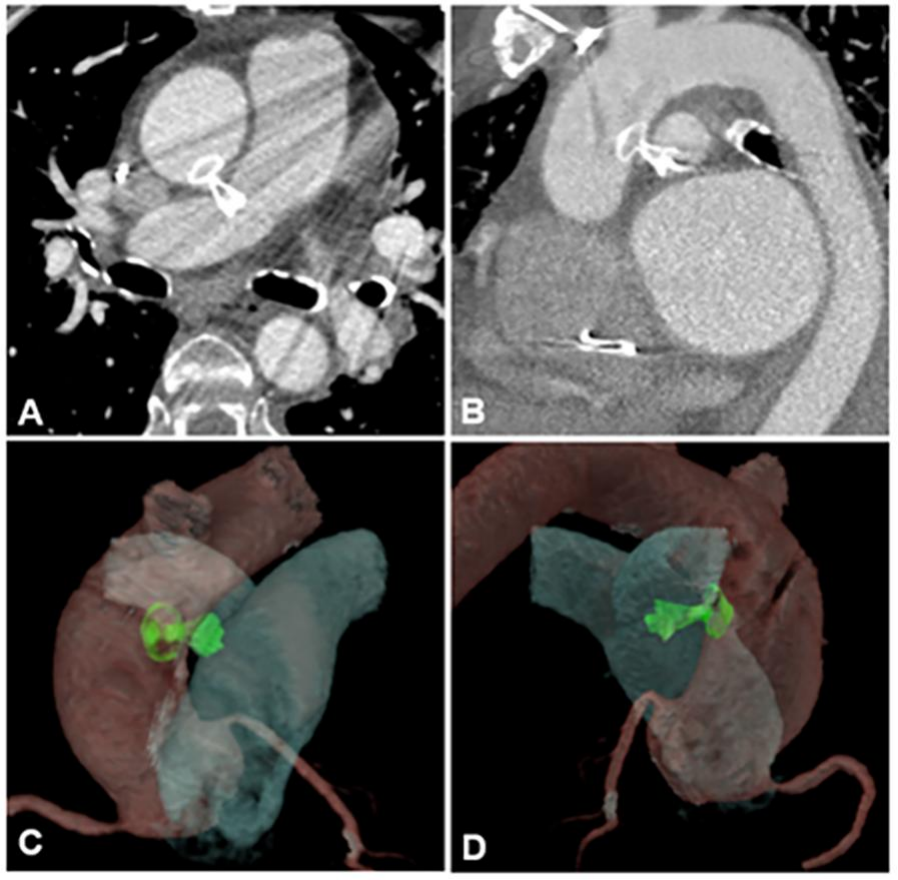
Images of aortic and pulmonary vessels angiotomography **(A,B)** performed after aortopulmonary fistula correction procedure, during the follow-up and its 3D reconstruction **(C,D)**.

During the 9-month follow-up period, the patient demonstrated significant clinical improvement with resolution of symptoms. A follow-up CTA of the aortic and pulmonary vessels confirmed the correct position of the device (Figures 3A–D), with a slight protrusion into the lumen of the proximal right pulmonary artery. TEE showed no evidence of pulmonary artery flow obstruction, pressure gradients, or hemodynamic compromise.

## 2 Discussion

Aortopulmonary fistula is a rare condition among ascending aortic pathologies, characterized by elevated pulmonary artery pressure, increased blood flow, and enhanced pulmonary volume. Its primary clinical manifestation is heart failure, typically presenting with fatigue, dyspnea, and, in more severe cases, acute pulmonary edema (1–3).

The leading causes include aortic aneurysm, acute aortic dissection, surgical manipulation, endocarditis, and trauma (1, 2, 4–7). Diagnosis can be established using transesophageal echocardiography, computed tomography angiography, or magnetic resonance imaging (1, 5, 8). Management of this condition is challenging and must be individualized to determine the most appropriate intervention, either percutaneous or surgical (1, 3, 9, 10).

Our patient underwent mitral valve replacement with emergency ascending aortic cannulation, which caused trauma to the aortic wall and subsequently led to the development of an aortopulmonary fistula. In this case, the decision to pursue percutaneous closure was made because it represented a less invasive alternative, and the patient had already undergone two prior sternotomies, posing a very high surgical risk. It should be noted, however, that percutaneous intervention also carries potential risks, including proximity of the device to the left coronary artery (not observed in our case), residual shunt, and vascular complications.

The tunnel-like course of the aortopulmonary fistula in this case supported the use of a PDA occlusion device. Other devices were considered; however, concerns were raised regarding their ability to achieve adequate sealing and maintain coaxial alignment. The selected device was successfully deployed, achieving complete closure of the fistula with no residual leak. Although a slight protrusion of the device into the pulmonary artery was observed, no hemodynamic consequences were noted. If such repercussions had occurred, an increased risk of thrombosis would need to be considered, and extended anticoagulation or antiplatelet therapy would have been warranted.

Given the rarity of this condition, no clinical trials comparing the available correction techniques have been published to date. The existing literature consists mainly of a few case reports that describe the diagnostic challenges, imaging modalities, and treatment approaches—either surgical or percutaneous. These reports do not demonstrate the superiority of any particular correction method. Furthermore, only a few cases of percutaneous closure of aortopulmonary fistula using occlusion devices have been reported in the literature (11–13). This statement is based on a review of high-impact publications indexed in PubMed.

However, regardless of the chosen approach, treatment requires advanced technical expertise and should be performed in specialized centers. Given the limited number of studies comparing surgical and percutaneous correction of secondary aortopulmonary fistula, as well as the scarcity of long-term follow-up data, further research is warranted.

Thus, the device should be selected based on the fistula size, morphology, and its proximity to the coronary arteries, atrioventricular valve, and pulmonary valve. The choice of prosthesis may also be influenced by device availability and supplier constraints. Follow-up should be performed at appropriate intervals to assess for device thrombosis, residual shunt, or pulmonary hypertension in the long term. We suggest performing an imaging study, such as CTA or TEE, at 6 months and continuing with annual follow-up if no warning signs are present. In conclusion, percutaneous closure represented an appropriate therapeutic option in this case, yielding excellent clinical and procedural outcomes.

## Data Availability

The raw data supporting the conclusions of this article will be made available by the authors, without undue reservation.
